# Self-Objectification, Disordered Eating and Sexual Orientation in Men

**DOI:** 10.3390/ijerph21010106

**Published:** 2024-01-17

**Authors:** Rachel Bachner-Melman, Lilac Lev-Ari, Hadar Tiram, Ada H. Zohar

**Affiliations:** 1 Clinical Psychology Graduate Program, Ruppin Academic Center, Emek Hefer 4025000, Israel; ldlevari@gmail.com (L.L.-A.); htiram2@gmail.com (H.T.); adaz@ruppin.ac.il (A.H.Z.); 2 School of Social Work, Hebrew University of Jerusalem, Jerusalem 9190500, Israel; 3 Lior Tsfaty Suicide and Mental Pain Research Center, Ruppin Academic Center, Emek Hefer 4025000, Israel

**Keywords:** sexual orientation, disordered eating, self-objectification, depressive symptoms, men

## Abstract

The interplay between disordered eating, depressive symptoms and self-objectification differs between genders and sexual orientations, and merits further study in homosexual and heterosexual men. We examined disordered eating, depressive symptoms and self-objectification in a sample of Israeli heterosexual and homosexual men. Participants were 215 men aged 19–65, 108 of whom were classified by the Kinsey scale as being heterosexual and 107 as homosexual. They completed online measures of self-objectification, disordered eating and depressive symptoms. Heterosexual men reported lower levels of disordered eating and self-objectification than homosexual men, however the difference in depressive symptoms was not statistically significant. Correlations between disordered eating, self-objectification and depressive symptoms when controlling for age, BMI and number of children were all significant, with similar patterns of association for heterosexual and homosexual men. Self-objectification partially mediated the association between sexual orientation and disordered eating. However, contrary to our hypothesis, sexual orientation (homosexual/heterosexual) did not moderate the association between disordered eating and self-objectification. The tendency of homosexual men towards self-objectification is linked to unhealthy eating habits. Self-objectification helps explain the propensity of homosexual versus heterosexual men to develop disordered eating and possibly eating disorders. It should therefore be targeted in prevention and in therapy.

## 1. Introduction

Young children develop a concept of their body and integrate it into their emergent self-image. The nascent body image is originally based on perceptions, and only later flavored by judgement. Even young children in pre-school acquire a bias against overweight [1] and this bias may be internalized by children who perceive themselves to be overweight, lowering their self-esteem and confidence. By the beginning of school, girls aspire to be thin and boys to be lean, muscular and tall [1]. If children perceive their bodies as meeting these internalized ideals, their body concept contributes to their self-esteem and confidence. Participating in organized sports contributes to children’s positive body image and self-esteem [2,3]. Teasing children about their body shape or weight has immediate [4] and long-term detrimental effects on body image and self-esteem [5]. Children as young as seven years of age who perceive themselves to be overweight practice dietary restraint, which in turn contributes to risk for a future eating disorder [6]. Comments about body shape and weight by others, whether positive, negative or neutral, contribute to a tendency to view one’s body as an object, or in other words, “self-objectification”. Self-objectification may enhance self-esteem, as in the case of the actor Paul Newman, nicknamed “the body”, who often removed his shirt on screen to show his well-shaped torso [7]; this could also be thought of as body narcissism [8], i.e., gaining attention and admiration by showing off one’s body. However, most people perceive gaps between their body and the cultural body ideal, so that self-objectification is usually a negative process related to low self-esteem, and incur vulnerability to develop and eating, depressive or anxiety disorder.

The process of building one’s identity while discovering male homosexuality feeds the process of self-objectification [9]. Grindr©, a male homosexual dating app, offers a detailed description of body types. Objective measurements such as height and weight are included, but users can also define themselves as members of 12 tribes. These tribes are modes of self-presentation (https://help.grindr.com/hc/en-us/articles/4402336949523-How-to-build-your-profile, accessed on 23 December 2024) that relate to body shape, body hair, penis size, and other aspects of identity and lifestyle choices. Self-objectification, identity, and desirability are therefore intricately interwoven.

Clinical eating disorders are characterized by problematic eating or eating-related behavior affecting the consumption or absorption of food and impairing physical health and/or psychosocial functioning [10]. The Diagnostic and Statistical Manual of Mental Disorders-5 (DSM-5; [10]) defines several eating disorders, including anorexia nervosa, bulimia nervosa, binge eating disorder, avoidant/restrictive food intake disorder (ARFID), and other specified feeding and eating disorder (OFSED). Symptoms of these disorders, such as restricting, bingeing and purging, are often present to a varying extent in the general population but are often not serious enough to warrant the diagnosis of a fully blown eating disorder. Such subclinical symptomatology is called “disordered eating” [11], characterized by a tendency to display unhealthy eating behaviors, and is examined in this study in relation to self-objectification and sexual orientation in men.

Towards the end of the last century, Fredrickson and Roberts’ [12] seminal paper on objectification theory presented a feminist social psychological theory that explained how Western cultures objectify females. According to this theory, women and girls learn, via social processes, behaviors and interactions, to think of their body as a collection of separate parts and value their bodies’ appearance as the most important determinant of their self-worth, rather than its agency and functionality. Self-objectification occurs when the self is internalized as an object or collection of body parts. Whereas objectification theory [13] and self-objectification [14] have been expanded and widely tested, relatively little relevant research has addressed self-objectification, or the internalization of self as an object or collection of body parts, in males. Nevertheless, self-objectification is clearly manifested in men as well as women [15,16,17].

Fredrickson et al. [12] examined the connection between self-objectification and disordered eating and found these variables to be significantly correlated for women, but not for men. This finding was subsequently replicated, with associations consistently observed between self-objectification and negative outcomes such as eating disorders [18], disordered eating [19,20] and depression [21] for women. Strelan and Hargreaves [17] found that both men and women with high levels of self-objectification had lower body self-esteem than men and women with low levels of self-objectification. Aubrey [22] found that media exposure was positively associated with self-objectification for women but not for men. Oehlhof et al. [16] explored the relationship between self-objectification and ideal body shape in men and women, with the goal of understanding body image issues in men. They observed an interaction between sex (male/female) and self-objectification for ideal body shape, so that women with high self-objectification desired a less muscular body whereas men with high self-objectification desired a more muscular body. Choma et. al. [23] found that objectification experiences elicited higher levels of body surveillance, shame and appearance anxiety in women than in men. These and similar studies would suggest that the relationship between self-objectification and other body-related variables tends to differ between men and women. However, results are not entirely consistent, and the connection of self-objectification to disordered eating and related variables in men merits further study.

Research on the connection between self-objectification and disordered eating has supported different correlational patterns of self-objectification for homosexual versus heterosexual men. Martins et al. [24] found significant differences between the levels of self-objectification, body shame, drive for thinness and body dissatisfaction reported by homosexual versus heterosexual men. In addition, they found that increasing self-objectification led to higher body shame, body dissatisfaction and restrained eating for homosexual but not for heterosexual men. Breslow et al. [25] found that the number of online dating apps used by sexual minority men was positively and significantly related to self-objectification. Naamani and El Jamil [26] found a significant and positive link between self-objectification and disordered eating in a sample of homosexual Lebanese men. Although research has indicated that lesbians, like heterosexual women, experience negative consequences of self-objectification [27], Noffsinger-Frazier [28] suggested that a lesbian identity protects women from self-objectification. This supports the notion that the male sexualized gaze is associated with self-objectification and that homosexual men may therefore be more prone to self-objectification than heterosexual men. Self-objectification theory may therefore be a useful framework for conceptualizing disordered eating and related body image disturbances in homosexual, but not heterosexual, men.

Another variable intricately tied to both self-objectification and to disordered eating is depression. According to Fredrickson and Roberts [29], self-objectification increases shame and anxiety about the body and appearance, which in turn contribute risk for eating disorders, sexual dysfunction and unipolar depression. A large body of research has unequivocally shown a strong relationship between the symptoms of eating disorders and of depression [30,31,32]. Self-objectification can increase risk for both these disorders. In a systematic review of quantitative studies investigating the connection between self-objectification and depression, Jones and Griffiths [33] reviewed 31 studies and found that self-objectification had a mediating effect on depressive symptoms in most studies with female participants. The two prospective studies included in this review [34,35] indicated a causal relationship between self-objectification and depression in women. However, the findings were inconsistent among men. Further research is therefore necessary to understand the underlying mechanisms linking self-objectification and depression in men.

### 1.1. Aim and Objectives

This study examines selflessness, disordered eating, depressive symptoms and self-objectification in a sample of heterosexual and homosexual men in Israel. Specifically, we wished to examine whether self-objectification and depression mediate the connection between sexual orientation and disordered eating for men. Since an association between self-objectification and disordered eating has been generally found to be stronger and more consistent among females than in males, as well as among homosexual men than in heterosexual men, we also aimed to examine whether sexual orientation moderates the relationship between self-objectification and disordered eating for men. In Israel, the level of religiosity is associated with early marriage, number of children, and reticence to openly expose a homosexual identity, so that we felt it important to include this variable.

### 1.2. Hypotheses

1. Heterosexual men will report lower levels of disordered eating, self-objectification and depressive symptoms than homosexual men.
2. Significant, positive correlations will be observed between disordered eating, self-objectification and depressive symptoms in the entire sample.
3. The association between sexual orientation (homosexual vs. heterosexual) and disordered eating will be mediated by self-objectification, depressive symptoms and BMI.
4. Sexual orientation will moderate the association between disordered eating and self-objectification.

## 2. Materials and Methods

### 2.1. Participants

A total of 215 men, aged 19–65 (M = 32.42, SD = 8.0), participated in the study and completed self-reported questionnaires. They included 108 heterosexual men and 107 homosexual men, recruited via a B.A. introductory psychology course at Ruppin Academic Center, by word of mouth and via social media (Facebook, Instagram, etc.), targeting both the general population and the LGBTQ+ community. Students at the Ruppin Academic Center received class credit and the other participants were non-paid volunteers. Five men who self-reported as bisexual and scored 3 on the Kinsey scale (equally heterosexual and homosexual) were excluded from the study, since this group was too small for any meaningful analyses. A further five respondents, who self-reported as bisexual categorically were classified as heterosexual because they endorsed either 1 (predominantly heterosexual, only incidentally homosexual) or 2 (predominantly heterosexual, but more than incidentally homosexual) on the Kinsey scale (see below). Three respondents who self-reported as bisexual categorically were classified as homosexual because they endorsed 4, predominantly homosexual, but more than incidentally heterosexual, on the Kinsey scale. Most of the participants (96.3%) were born in Israel.

### 2.2. Instruments

#### 2.2.1. Demographic Variables

Participants reported their age, place of birth, education, level of religiosity, height, weight, relationship status and years of education.

#### 2.2.2. Sexual Orientation

Sexual orientation was assessed using a modified version of the Kinsey scale [36] that assessed sexual attraction to people of the same and/or opposite sex according to sexual fantasies, or sexual arousal when thinking about men and/or women. Responses were rated on a 7-point scales on a continuum between 0 (exclusively homosexual) and 6 (exclusively heterosexual). In this study participants completed a Hebrew version previously used in research [37]. Cronbach’s alpha of the English version is 0.89 and in this study it was 0.95.

#### 2.2.3. Self-Objectification

Self-objectification was measured using the Likert version of the 10-item Self-Objectification Questionnaire (LSOQ; [38]), a revised version of the original Self-Objectification Questionnaire [19]. The LSOQ assesses the extent to which participants view themselves as objects evaluated for their appearance, rather than as a human being with various competencies. Respondents are asked to indicate (from 1 = extremely low impact to 11 = extremely high impact) the extent to which various body attributes impact their physical self-concept. Five attributes are based on body appearance (i.e., weight, sex appeal, physical attractiveness, firm/sculpted muscles and measurements), and five on body competence (i.e., strength, physical coordination, energy level, health and physical fitness), yielding an appearance score and a competence score. Since these did not differ significantly, the LSOQ score was calculated as a simple mean of all ten items. The LSOQ was translated into Hebrew for the purpose of this study via translation, backtranslation and adjustment. Wollast et al. [38] found a Cronbach alpha of 0.80 and in this study it was 0.82.

#### 2.2.4. Disordered Eating

Disordered eating was assessed using the 13-item Eating Disorder Examination Questionnaire (EDE-Q13; [39]), a reliable and valid short version of the 28-item EDE-Q [40] that enquires about problematic eating attitudes and behaviors over the previous 28 days. The EDE-Q comprises five subscales: eating restraint (3 items), shape and weight concerns (2 items), body dissatisfaction (2 items), bingeing (3 items) and purging (3 items). Responses are noted on a 7-point ordinal response, with higher scores reflecting more disordered eating. A sample item is: “On how many of the past 28 days have you been deliberately trying to limit the amount of food you eat in order to influence your shape or weight (whether or not you have succeeded)?” The Cronbach’s alpha values reported by Lev-Avi et al. [39] for the subscales of EDE-Q-13 were reported as 0.99 for SWO, 0.89 for BD, 0.92 for ER, 0.89 for bingeing and 0.63 for purging. In this study we used the original Hebrew version [39] that yielded an overall Cronbach alpha of 0.88.

#### 2.2.5. Depressive Symptoms

Depressive symptoms were measured using the 9-item, single-factor Patient Health Questionnaire (PHQ-9; [41]) that assesses diagnostic criteria for a major depressive episode from the DSM-5 [10]. Respondents are asked how often, over the past two weeks, they have been bothered by various symptoms, e.g., “Little interest or pleasure in doing things”. Responses are recorded between 0 (not at all) to 3 (nearly every day), with higher scores indicating more symptoms. The PHQ-9 has been found to be valid for use in both clinical and non-clinical populations [42,43]. A Hebrew version widely used in previous studies (e.g., [44]) was used in this study and yielded a Cronbach alpha of 0.88.

### 2.3. Procedure

Ethics approval was given by the Ruppin Academic Center Ethics Committee. The study was presented as examining social attitudes among men and women. Participants received an online link to the questionnaires and completed them via Qualtrics (www.qualtrics.com). After providing informed consent on the first screen, they answered demographic questions, including on the Kinsey scale, followed by the questionnaires listed above, presented in random order to minimize order effects.

Data were analyzed using SPSS 28. Scores were compared between heterosexual and heterosexual men using MANOVA. All variables were assessed for normality and were found to be normally distributed. Pearson correlations between study variables were calculated and compared between groups using Fisher’s Z test. To test whether self-objectification and depression mediated the association between sexual orientation and disordered eating, we used a mediation model based on PROCESS V3.5 model 4. PROCESS (model 1) was also used to assess the moderation hypothesis.

## 3. Results

Preliminary analyses were conducted to identify potential covariates of the study variables by examining between-group demographic differences via chi-square tests (relationship status and religiosity) and MANOVA (age, height, weight, BMI, years of education and number of children). The findings are presented below in Table 1 and Table 2. The MANOVA was statistically significant (F_(6,207)_ = 7.85, *p* < 0.001). As expected, religiosity was associated with heterosexuality. There were significant between-group differences for all variables except education and mother’s education. Age, BMI and number of children were therefore held constant in the analyses.

**Hypothesis** **1:** *Heterosexual men will report lower levels of disordered eating (EDE-Q-13), self-objectification (LSOQ) and depressive symptoms (PHQ-9) than homosexual men*.

**Figure 1:**
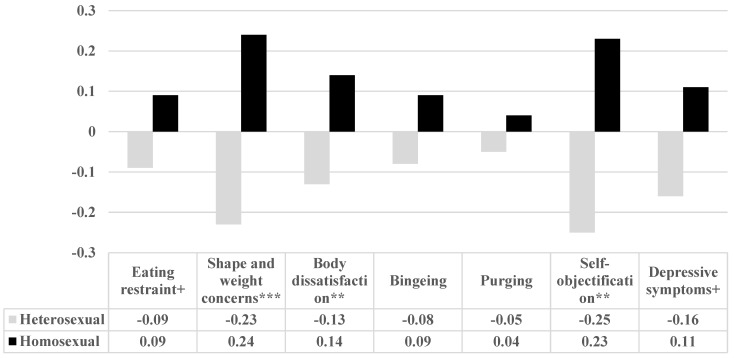
Differences between homosexual and heterosexual men for disordered eating (EDE-Q-13), self-objectification (LSOQ) and depressive symptoms, while controlling for age, BMI and number of children. Note: + *p* < 0.10, ** *p* < 0.01, *** *p* < 0.001.

In order to test this hypothesis, a MANCOVA was run with sexual orientation (heterosexual vs. homosexual) as the independent variable and disordered eating, self-objectification and depressive symptoms as the dependent variables. Age, BMI and number of children were held constant. The overall model was statistically significant (F_(7,187)_ = 2.82, *p* = 0.008) and between-group differences between the two groups are shown in Figure 1.

Eating restraint, shape and weight concerns, body dissatisfaction, bingeing and purging were measured by the EDE-Q-13, self-objectification by the LSOQ and depressive symptoms by the PHQ-9.

Figure 1 shows that homosexual men scored higher than heterosexual men on all study variables and that these differences reached statistical significance for shape and weight concerns (EDE-Q-13), body dissatisfaction (EDE-Q-13) and self-objectification (LSOQ). The total score for disordered eating (EDE-Q-13) was also statistically significantly higher for homosexual men (mean = 0.19, SD = 1.01) than for heterosexual men (mean = −0.19, SD = 0.96); (F_(1,198)_ = 11.61, *p* < 0.001).

**Hypothesis** **2:** *Significant, positive correlations will be observed between disordered eating (EDE-Q-13), self-objectification (LSOQ) and depressive symptoms (PHQ-9) in the entire sample*.

Table 3 shows Pearson correlations depicting the associations between disordered eating, self-objectification and depressive symptoms when controlling for age and number of children. All correlations were significant at *p* < 0.001, with the exception of that between eating restraint (EDE-Q-13) and depressive symptoms (PHQ-9), which was significant at *p* < 0.05, and those between self-objectification (LSOQ), bingeing and purging (EDE-Q-13), which were not significant. BMI was positively and significantly correlated with depressive symptoms (PHQ-9) but its association with self-objectification (LSOQ) did not reach significance. For a full correlation table, see Appendix A (Table A1). When Fisher’s Z tests were used to compare correlations between heterosexual and homosexual men, no significant differences were observed.

**Hypothesis** **3:** *The association between sexual orientation (homosexual vs. heterosexual) and disordered eating (EDE-Q-13) will be mediated by self-objectification (LSOQ), BMI and depressive symptoms (PHQ-9)*.

**Figure 2:**
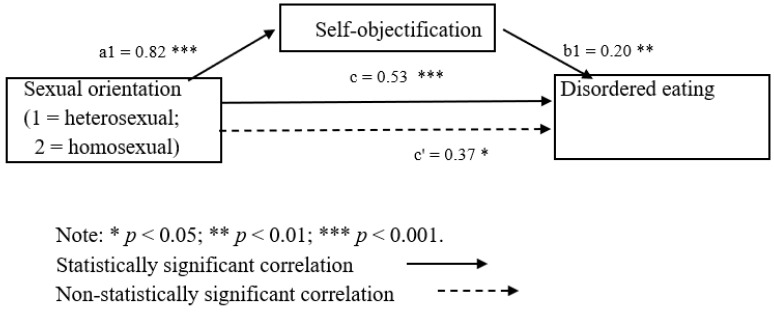
Mediation model of self-objectification and depressive symptoms on the association between sexual orientation and disordered eating.

We entered self-objectification (LSOQ) and BMI, but not depressive symptoms (PHQ-9) scores, into the mediation analysis, since the association between depressive symptoms and sexual orientation was not statistically significant, and significance of all associations is a precondition for testing mediation [45]. To test whether self-objectification (LSOQ) and BMI mediated the association between sexual orientation and disordered eating (EDE-Q-13) when controlling for age and number of children, a bootstrap analysis was employed. As advised by Hayes [45], 5000 bootstrap samples were used, using PROCESS V3.5 (see Figure 2). We first examined the association between sexual orientation and disordered eating (EDE-Q-13), which was significant (β = 0.44, *t* = 2.54, *p* = 0.01). The association between sexual orientation and self-objectification (LSOQ) was also significant (β = 0.76, *t* = 2.98, *p* < 0.001). We then examined whether self-objectification (LSOQ) mediated the association between sexual orientation and disordered eating (EDE-Q-13). When self-objectification (LSOQ) was entered as a mediating variable, the correlation between sexual orientation and disordered eating (EDE-Q-13) decreased (β = 0.37, *t* = 2.34, *p* = 0.02). The confidence interval for indirect pathway was calculated. The mediation was statistically significant [0.04, 0.29]. Self-objectification was therefore found to partially mediate the association between sexual orientation and disordered eating (EDE-Q-13). BMI, however, was not found to mediate the association between sexual orientation and disordered eating (EDE-Q-13).

**Hypothesis** **4:** *Sexual orientation (homosexual vs. heterosexual) will moderate the association between disordered eating (EDE-Q-13) and self-objectification (LSOQ)*.

**Figure 3:**
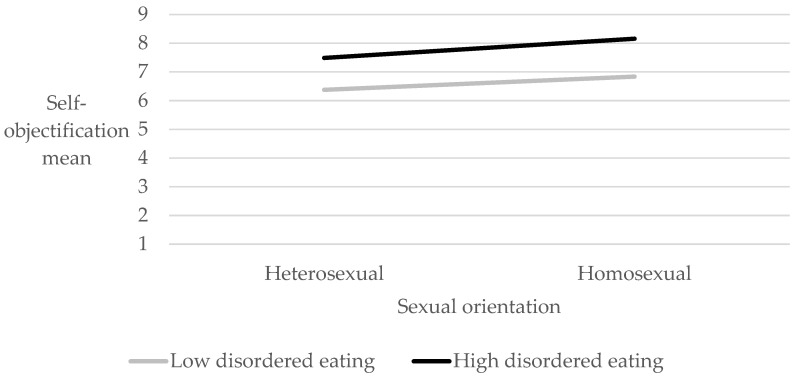
Moderation model of sexual orientation on the association between disordered eating and self-objectification.

To test whether sexual orientation (homosexual/heterosexual) moderated the association between disordered eating (EDE-Q-13) and self-objectification (LSOQ) when controlling for age, BMI and number of children, a bootstrap analysis was employed. As advised by Hayes [45], 5000 bootstrap samples were used, using PROCESS V3.5. The interaction was not statistically significant, and the moderation hypothesis was therefore not confirmed. As can be seen in Figure 3, men high in disordered eating were higher in self-objectification, regardless of their sexual orientation.

## 4. Discussion

This study examined connections between self-objectification, disordered eating, depression and sexual orientation in a community sample of Israeli men. As expected, homosexual men scored significantly higher than heterosexual men on self-objectification. This might be a consequence of the developmental process in which male homosexual orientation and body image co-develop in childhood and adolescence and are coded into a dozen potential tribes, as codified in the tribes feature of the app Grindr©. This finding is in line with past research, which has consistently shown that homosexual men score higher than heterosexual men on a range of self-objectification measures [24,26,46,47]. It has long been claimed that homosexual men live in a subculture that objectifies the body and places great emphasis on physical attractiveness [48]. The finding that homosexual men scored higher than heterosexual men on self-objectification supports the clear emphasis they place on their and potential sexual partners’ physical characteristics, such as body type and appearance [49]. It supports an extension of objectification theory [24], according to which homosexual men experience the male gaze similarly to women, internalize this objectified view, and invest in maintaining a pleasing appearance to other men. Furthermore, the high levels of self-objectification we observed in homosexual men are in line with minority stress theory [50]. Minority stress theory suggests that sexual minority individuals encounter distinctive hardships due to their minority status, which exposes them to stigma, prejudice and discrimination, negatively impacting their mental health. This theory provides a widely accepted explanation for the higher prevalence of health problems in general [51,52] and disordered eating specifically [53] among sexual minority versus heterosexual individuals.

Another expected finding was that homosexual men scored significantly higher than heterosexual men on all facets of disordered eating. This difference was driven mainly by body dissatisfaction, as well as by shape and weight concerns. The finding that homosexual men have higher rates of disordered eating than heterosexual men has been frequently and consistently established, as can be seen from a pertinent literature review by Parker and Harriger [53]. According to objectification theory [29], as expanded to include men [24], it is important to understand that homosexual men are at risk for eating pathology, as are heterosexual women, because disordered eating is one of the corollaries of self-objectification.

The difference between homosexual and heterosexual men for depressive symptoms, however, was not statistically significant. Many studies [51,54], but not all [55], have found homosexual men to have higher levels of depressive symptoms than heterosexual men. The small sample in this study and resulting lack of statistical power may provide the best explanation, since the between-group comparison on depressive symptoms in fact approached significance. However, the Patient Health Questionnaire may be less appropriate for the assessment of depressive symptoms in non-clinical samples such as that in this study than for the assessment of the severity of clinical depression.

All correlations between study variables (disordered eating, self-objectification, depressive symptoms, BMI) were significant, with the exception of the correlation between depressive symptoms and self-objectification, that between BMI and purging and that between BMI and self-objectification (LSOQ). According to objectification theory [29], objectifying oneself contributes to depressive symptoms in females, and self-objectification and depressive symptoms have indeed been consistently found to be associated in women. A systematic review of studies on self-objectification and depression [33] reported that 27 of the 28 studies incorporating data from female participants observed a significant association between depression and self-objectification or the closely related concept of self-surveillance. Findings are, however, far less consistent among men, with some studies observing a significant correlation [21,55] and others not [34,56]. Serpa [55] found a direct association between self-objectification and depression in men, whereas others found that this association was mediated by body shame [57,58], appearance anxiety [20,21], body dissatisfaction [21] or flow [20]. Our finding of a non-significant association between self-objectification and depressive symptoms in men replicates some previous findings [37,56,59]. Clearly, the relationship between self-objectification and depressive symptoms in homosexual men is worthy of further investigation.

Depressive symptoms were found to be significantly associated with overall disordered eating, as well as with all five disordered eating subscales in our study. This association between disordered eating and depressive symptoms has been widely and consistently reported in different genders, ages, sexual orientations and cultures [60,61,62,63]. Both disordered eating and depressive symptoms increase during adolescence [64] and predict later mental health problems [65,66]. Evidence points to shared genetic factors between the two [67].

Self-objectification was found to be significantly associated with disordered eating in our study, supporting previous research [68]. Martins et al. [24] found that following experimental manipulation of state self-objectification, homosexual but not heterosexual men experienced an increase in dietary restraint. Therefore, although this study was purely correlational, it seems likely that disordered eating is a consequence rather than a cause of self-objectification. In terms of subscales, self-objectification scores were significantly linked to eating restraint, body dissatisfaction, and shape and weight concerns, but not to bingeing or to purging. Little research has addressed the relationship between self-objectification and bulimic symptoms (bingeing and purging), so that the lack of association we found between these variables should be replicated in future investigations.

Notably, there were no significant between-group differences in any of the correlational patterns observed. It is of specific interest that the correlation between self-objectification and disordered eating did not differ significantly between homosexual and heterosexual men. This is because our study did not replicate previous studies in which sexual orientation (homosexual/heterosexual) moderated the association between disordered eating and self-objectification among males [24,69,70]. A possible moderating role for sexual orientation in the relationship between disordered eating and self-objectification should therefore continue to be explored in future research.

However, self-objectification was found to partially mediate the association between sexual orientation and disordered eating (EDE-Q-13) in our sample of Israeli men. The tendency of homosexual men towards self-objectification therefore contributes to their well-established difficulties in the realm of eating attitudes and behaviors. Another variable that has previously been found to mediate the association between sexual orientation and disordered eating in men is susceptibility to social messages [37]. The internalization of social messages is intricately connected to the internalization of objectification. Society in general, and homosexual male subculture in particular, objectifies the bodies of women and homosexual men, and this view is constantly conveyed in myriad ways via the visual media. One outcome is the internalization by homosexual men of a view of their body as a collection of parts, a sexual object, rather than a whole person. In this study, this self-objectification partially explained the connection between being homosexual and having problematic eating attitudes and behaviors. Other mediators, or factors that contribute to the complex range of factors that tie male sexual orientation to disordered eating, should be identified in future research.

This study has its limitations. The sample was a convenience sample and was relatively small, limiting statistical power and possibly the significance of results. Participants were primarily young, Jewish Israeli men so that conclusions cannot necessarily be extended to other ages and cultures. They were also non-clinical community volunteers with relatively low levels of eating disorder and depression symptoms, so results cannot be generalized to people with clinically diagnosable eating disorders and depression. Older men were included in the sample, which may have affected outcomes relevant to body image, self-objectification and/or disordered eating. Data on sociosexuality, sexual behavior and number of sexual partners were not collected. The study relied on self-report and is thus subject to bias on that score. It was also cross-sectional, with data collected at a single point in time, limiting our ability to reach conclusions about the chronological emergence of the characteristics studied. Future research should examine whether findings from this study extend to minorities other than homosexual men.

In terms of clinical implications, self-objectification seems closely linked to disordered eating in both homosexual and heterosexual men and should therefore be addressed in eating disorder prevention programs and in psychotherapy for disordered eating. Prevention programs could focus on media literacy to critically evaluate media messages about masculinity and body image and promote body positivity, health, well-being and self-acceptance. Participants could be encouraged to challenge stereotypes within the LGBTQ+ community and wider society, and to seek support from friends, groups or communities that provide a sense of belonging and reduce the need for external validation based on appearance. In individual psychotherapy, men could be helped to develop multiple aspects of self-worth beyond physical appearance and explore talents, hobbies, skills, and personal achievements to build self-esteem. Mindfulness and self-compassion could help men to foster a kinder relationship with themselves more aware of their thoughts and emotions. Since homosexual men experience higher levels than heterosexual men of both self-objectification and disordered eating, efforts to decrease self-objectification in this population seem particularly important.

## 5. Conclusions

In accordance with objectification theory [29], homosexual men, like heterosexual women, are commonly seen by men as sexual objects and are therefore particularly vulnerable to internalizing this perspective (self-objectification), to viewing their bodies as sexual objects and adopting a sexist male view of themselves. This tendency towards self-objectification is linked to unhealthy eating habits that may form part of a maladaptive strategy to make themselves attractive to other men. Self-objectification is one of the factors that helps explain the propensity of homosexual as opposed to heterosexual men to develop disordered eating and possibly fully blown clinical eating disorders. Measures should be taken on the sociocultural, family and individual levels to help men become more aware of their tendency to self-objectify and combat it as far as possible. Decreased levels of self-objectification are likely to promote physical and psychological self-acceptance and counter disordered eating attitudes and behaviors.

## Figures and Tables

**Table 1:** Descriptive characteristics of the study groups (continuous).

Variable	Heterosexual (*n* = 108) Mean (SD)	Homosexual (*n* = 107) Mean (SD)	F_(1,212)_
Age	33.59 (8.96)	31.27 (6.75)	4.56 *
BMI	25.08 (3.96)	23.75 (3.24)	7.23 **
Education (years)	14.03 (3.04)	14.38 (3.03)	0.73
Mother’s education	13.75 (3.17)	13.05 (3.41)	2.42
Father’s education	13.51 (4.07)	12.26 (4.11)	5.02 *
Number of children	0.85 (1.26)	0.11 (0.50)	31.50 ***

* *p* < 0.05; ** *p* < 0.01; *** *p* < 0.001.

**Table 2:** Descriptive characteristics of the study groups (categorical).

Variable	Heterosexual (*n* = 108) N (%)	Homosexual (*n* = 107) N (%)	$\chi^{2}$
Relationship status			
Not in a relationship	37 (35.9%)	56 (53.8%)	
In a relationship	37 (35.9%)	41 (39.4%)	
Married	29 (28.2%)	7 (6.7%)	$\chi_{\left(2\right)}^{2}$ = 17.53 ***
Religiosity			
Secular	77 (71.3%)	87 (81.3%)	
Traditional	17 (16.2%)	12 (11.9%)	
Orthodox	11 (10.5%)	2 (2.0%)	$\chi_{\left(2\right)}^{2}$ = 7.63 *

* *p* < 0.05; *** *p* < 0.001.

**Table 3:** Pearson correlations between disordered eating (EDE-Q-13), self-objectification (LSOQ) and depressive symptoms (PHQ-9), controlling for age and number of children.

		Self-Objectification (LSOQ)	Depressive Symptoms (PHQ-9)
Disordered eating (EDE-Q-13) total		0.35 ***	0.40 ***
	Eating restraint	0.26 ***	0.20 **
	Shape and weight concerns	0.42 ***	0.35 ***
	Body dissatisfaction	0.27 ***	0.31 ***
	Bingeing	0.16	0.39 ***
	Purging	0.11	0.30 ***
Self-objectification (LSOQ)			0.15 *
BMI		0.11	0.17 *

Note: * *p* < 0.05, ** *p* < 0.01, *** *p* < 0.001. LSOQ = Likert version of the Self-Objectification Questionnaire; EDE-Q-13 = Eating Disorders Examination—Questionnaire-13; PHQ-9 = Patient Health Questionnaire-9; BMI = Body mass index.

## Data Availability

The data on which this study is based are available on request from the corresponding author (rachel.bachner@mail.huji.ac.il).

## Appendix A

**Table A1:** Pearson intercorrelations between study variables: Disordered eating (EDE-Q-13), self-objectification (LSOQ) and depressive symptoms (PHQ-9), controlling for age and number of children.

		2	3	4	5	6	7	8	9
Disordered eating (EDE-Q-13)	1. Total	0.85 ***	0.83 ***	0.73 ***	0.53 ***	0.36 ***	0.34 ***	0.37 ***	0.34 ***
	2. Eating restraint		0.64 ***	0.44 ***	0.28 ***	0.18 *	0.25 ***	0.17 *	0.18 *
	3. Shape and weight concerns			0.59 ***	0.20 **	0.16 *	0.40 ***	0.33 ***	0.22 **
	4. Body dissatisfaction				0.28 ***	0.11	0.25 ***	0.27 ***	0.32 ***
	5. Bingeing					0.34 ***	0.08	0.35 ***	0.41 ***
	6. Purging						0.10	0.29 ***	0.12
7. Self-objectification (LSOQ)								0.13	0.11
8. Depressive symptoms (PHQ-9)									0.17 *
9. BMI									

Note: * *p* < 0.05, ** *p* < 0.01, *** *p* < 0.001. LSOQ = Likert version of the Self-Objectification Questionnaire; EDE-Q-13 = Eating Disorders Examination—Questionnaire-13; PHQ-9 = Patient Health Questionnaire-9; BMI = Body mass index.
